# Supplementary material for: All-dielectric magnetic metasurface for advanced light control in dual polarizations combined with high-Q resonances
Source: Nat Commun. 2020 Oct 30;11:5487. doi: 10.1038/s41467-020-19310-x (PMC7599251; doi:10.1038/s41467-020-19310-x)
Supplement: Supplementary file 1 — Supplementary Information [file 41467_2020_19310_MOESM1_ESM.pdf]

All-dielectric magnetic metasurface for advanced light control in dual polarizations combined with high-Q resonances

Daria O. Ignatyeva[1,2,3*], Dolendra Karki[4], Andrey A. Voronov[1,3], Mikhail A. Kozhaev[2,3,5], Denis M. Krichevsky[2,3,6], Alexander I. Chernov[3,6], Miguel Levy[4], Vladimir I. Belotelov[1,2,3]

[1] Faculty of Physics, Lomonosov Moscow State University, Moscow, Russia

[2] Crimean Federal University, Simferopol, Russia

[3] Russian Quantum Center, Moscow, Russia

[4] Physics Department, Michigan Technological University, Houghton, Michigan, U.S.A.

[5] Prokhorov General Physics Institute of the Russian Academy of Sciences, Moscow, Russia

[6] Center for Photonics and 2D Materials, Moscow Institute of Physics and Technology (National Research University), Dolgoprudny Russia

Supplementary Information

Supplementary Note 1. The shape of the fabricated nanopillars.

Etching in phosphoric acid resulted in the tilting of the nanopliiar sidewalls so that their shape is close to the truncated cones. The magnified SEM image, schematic representation of the nanopillar parameters and AFM profiles with different scales are shown in Supplementary Figure 1. The estimated vertical angle, based on the average measurements of 128 profiles, is 21.3 ± 0.2 deg.

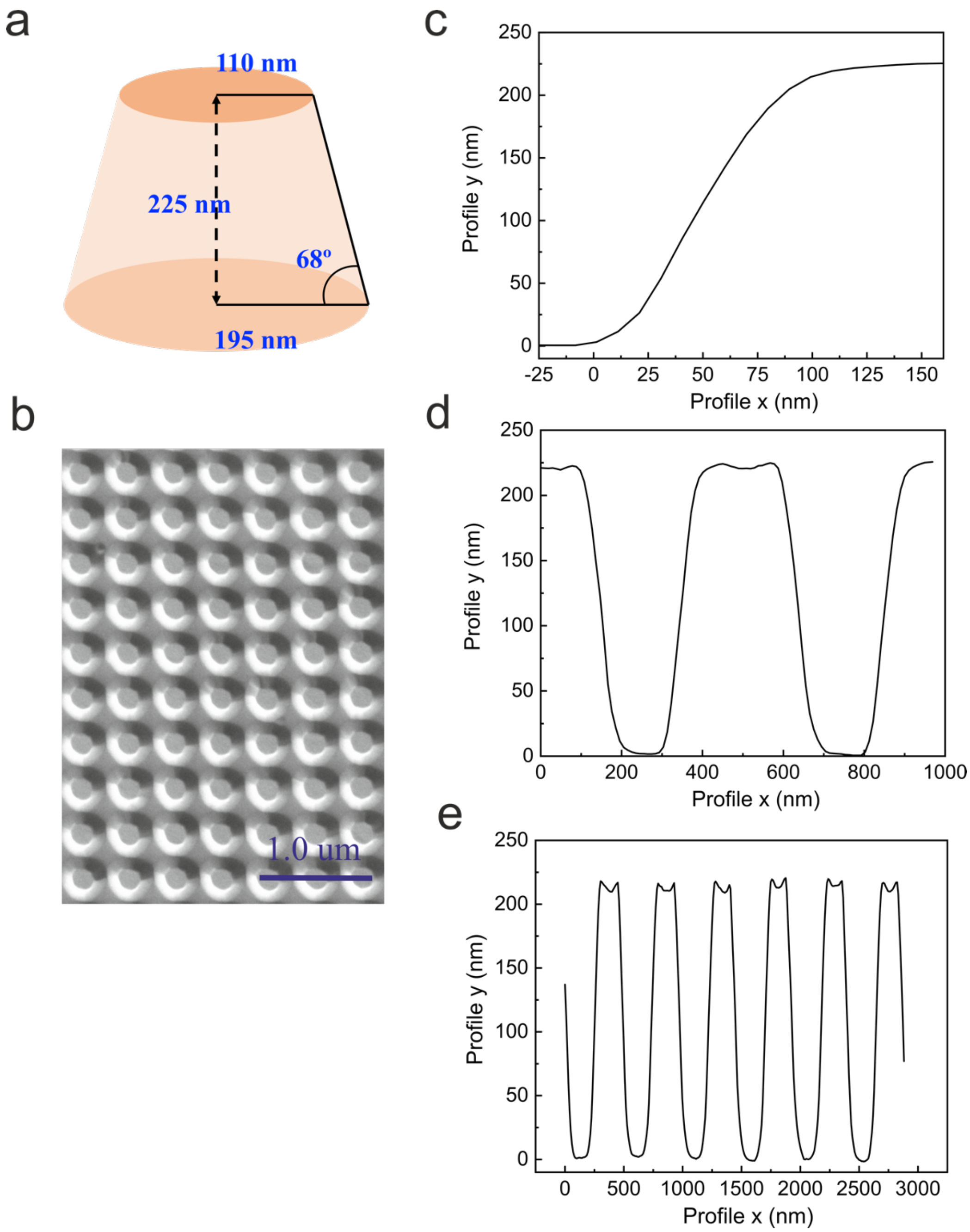

Supplementary Figure 1. Scheme of the nanopillars and magnified SEM image (left panel). AFM profiles of the nanopillars (right panel).

Atomic Force Microscopy (AFM) imaging was performed with a Bruker Multimode V8 device operating in the Peak Force-HR tapping regime with ScanAsyst HR probes. The tip side angle is 17.5 deg, tip radius is 2 nm.

Supplementary Note 2. The impact of nanopillar shape on guided modes.

The specific shape of the nanopillars make a very moderate affect on the process of the excitation of the guided modes. Supplementary Figure 2 shows the transmittance spectra for the nanopillars having the shape of the truncated cones vs. shape of the cylinders with similar volume. One may see a very moderate variation of the resonance positions.

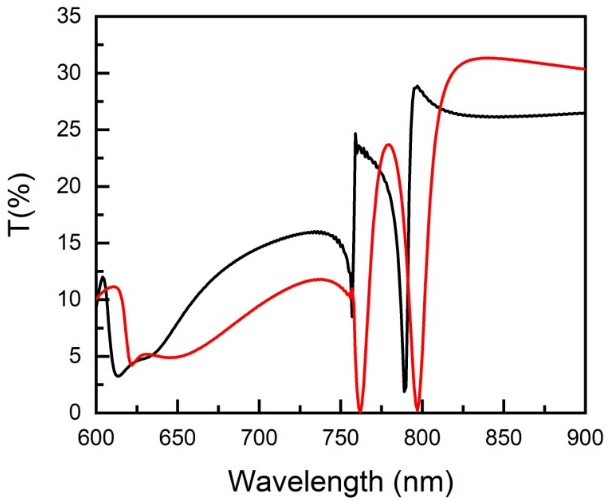

Supplementary Figure 2. The numerical transmittance spectra of the 2D structure with truncated cones (red) vs. cylinder (black).

The corresponding electromagnetic field distributions are also very similar in these structures.

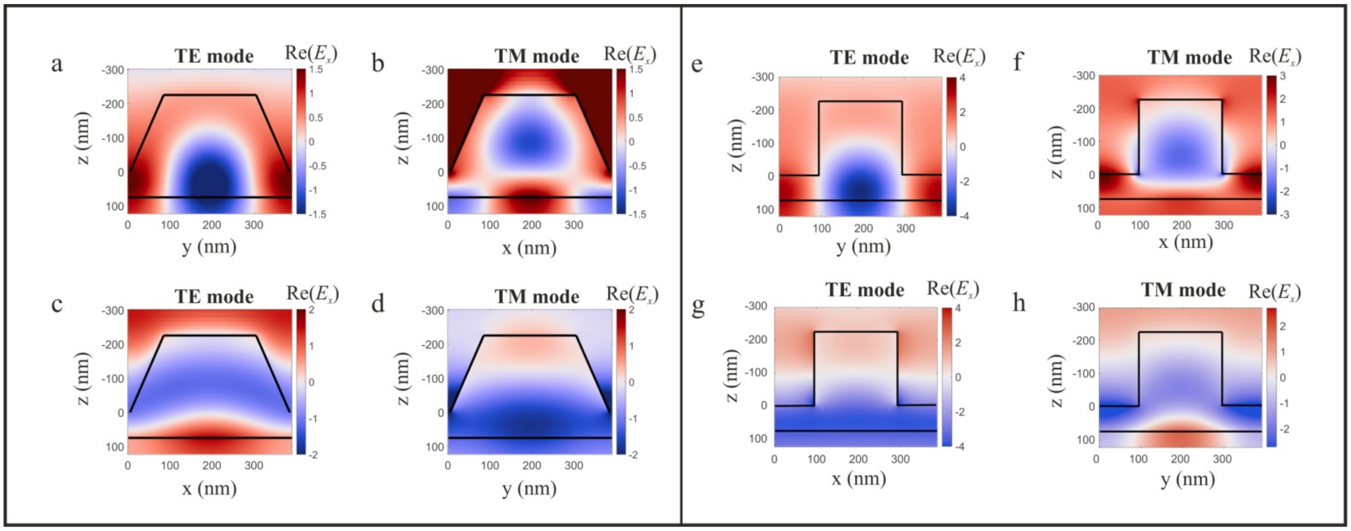

Supplementary Figure 3. Electromagnetic field distribution $\mathrm{Re}(E_\mathrm{x})$ for the TM- and TE-modes in the metasurface with truncated cone (left panel) and cylinder (right panel) shape of the nanopillars. The TE(0,1)-mode propagating in y-direction and the TM(1,0)- mode propagating in x- direction, excited by p-polarized light with $\mathbf{E} = (E_\mathrm{x}, 0, 0)$ at normal incidence are shown. All the cross-sections are taken at the center of the nanopillar. One period of magnetic metasurface is shown. Images show the field distribution in a normal to sample surface plane: upper panel images show the field distribution in the direction along the wave vector and bottom panel show the field distribution in a plane orthogonal to the wavevector of the modes.

Supplementary Note 3. Q-factor of the guided mode resonances.

There are 3 main channels of losses for a guided mode excited in the structure: 1) absorption in BIG material 2) leakage of radiation due to the back-coupling to the propagating reflected/transmitted waves 3) scattering losses due to the fabrication inaccuracies. As surface roughness is < 1 nm and the non-resonant transmittance in the transparency region is close to the one without nanostructure (reflection at the GGG substrate/air interface give $T = 80\%$ as the theoretical limit). Thus, one can neglect spurious scattering due to surface roughness 3) as a loss channel and assume

$$Q^{-1} = Q_\mathrm{abs}^{-1} + Q_\mathrm{leak}^{-1}. \tag{1}$$

Q_abs can be roughly estimated as $Q_\mathrm{abs} = n'_\mathrm{BIG}/n''_\mathrm{BIG}$ that gives $Q_\mathrm{abs} = 954$ at 800 nm (see the model of smooth BIG permittivity in Methods section which gives $n_\mathrm{BIG} = 2.5650 + 0.0028\mathrm{i}$). Thus, for experimentally observed $Q = 109$. (TE(1,0)-mode) one may get an estimation $Q_\mathrm{leak} = 123$. Thus, the main losses and Q-factor limitation comes from the back-coupling of the guided mode to the propagating radiation. This fact was confirmed by the numerical simulations: the resonance widths do not change much if all of the layers are treated as lossless, as shown in Supplementary Figure 4.

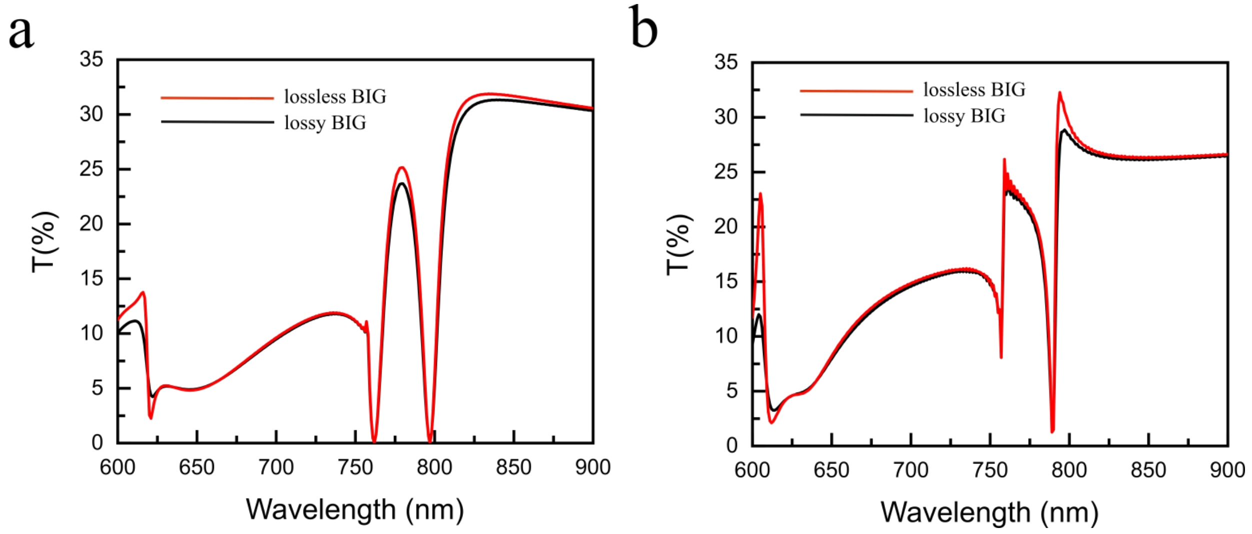

Supplementary Figure 4. Transmittance spectra for normal incidence for lossy (black color) and lossless BIG material of the nanopillars having the truncated cone (a) or cylinder (b) shape.

There is an intrinsic angular dependence of the resonance Q-factor. One may write (see Eq.(2)):

$$(k_0 \sin\theta + l_x G_x)^2 + (l_y G_y)^2 = (k_0 n'_\beta)^2$$

$$(k_w \sin\theta + l_x G_x)^2 + (l_y G_y)^2 = (k_w (n'_\beta + n_\beta''))^2 \qquad (2)$$

for the resonance wavelength λ_0 and for the wavelength corresponding to the resonance half-height λ_w (where $n_\beta = n'_\beta + i \cdot n''_\beta$ is the mode refraction index β/k_0). Assuming n_β independent on the wavelength in the considered region of $\Delta\lambda < 50$ nm near the resonance center and $\lambda_w - \lambda_0 \ll \lambda_0$ one may estimate the quality factor

$$Q = \frac{\lambda}{\Delta\lambda} = \frac{\lambda_0}{2(\lambda_w - \lambda_0)} = \frac{n'^2_\beta - \sin\theta(k_0 \sin\theta + m_x G_x)}{2 n'_\beta n_\beta''}, \qquad (3)$$

where θ should be taken with the sign corresponding to the mutual $\boldsymbol{\beta}$ and $\mathbf{k}_\tau^{\mathbf{inc}}$ propagation direction. Supplementary Figure 5 illustrates this dependence and its difference for (0,1) and (1,0) modes. The numerical estimations and experimental values of $Q(\theta = 0)$ and $Q(\theta = 44)$ are given in the table below.

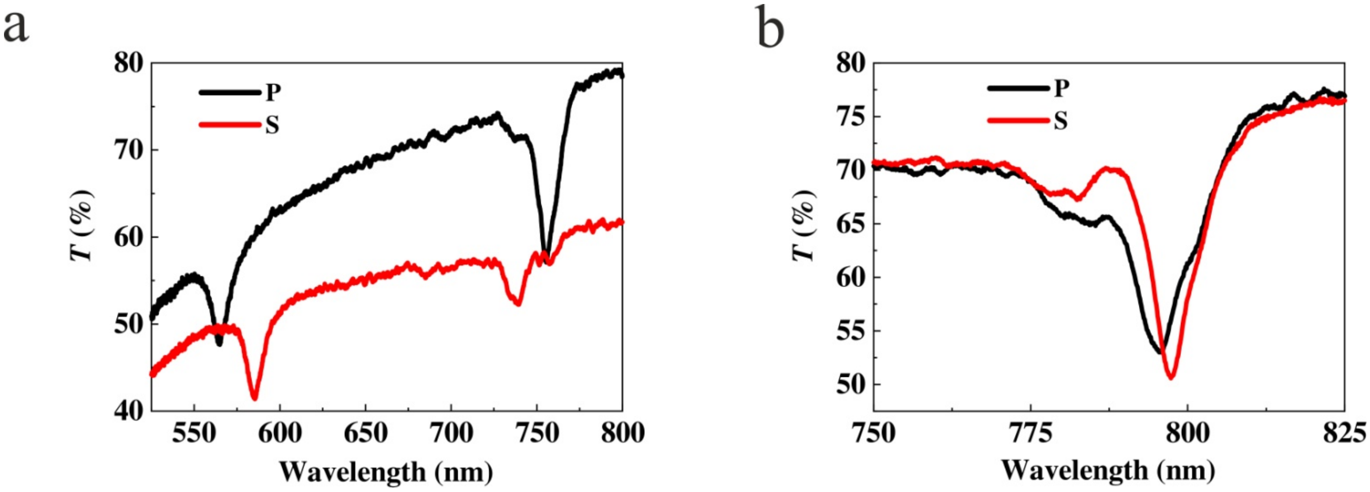

Supplementary Figure 5. Transmittance spectra for p- and s- polarized light at (a) $\theta = 0°$ and (b) $\theta = 44°$.

Supplementary Table 1. Numerical estimations and experimental values of Q-factor at the oblique incidence.

	TM(1,0)	TE(1,0)	TM(0,1)	TE(0,1)
$Q(\theta = 0)$ (exper.)	57	109	76	75
$Q(\theta = 44)$ (exper.)	43	67	70	58
$Q(\theta = 44)$ (estim.)	37	71	67	65

Supplementary Note 4. Numerical simulations of TMPIE.

Electromagnetic simulation of modes propagation inside the unit cell of the considered all-dielectric structure was carried out by numerical solution of Maxwell equations using the rigorous coupled-wave analysis (RCWA) approach [S1, S2]. Iron-garnet film was described by the following spectral dependence of permittivity (ε) and gyration constant (g):

$$\varepsilon(\lambda) = (0.1207 \cdot \lambda + 0.9119) \cdot \left(1.07 + \frac{4.90}{1 - \left(\frac{0.303}{\lambda}\right)^2} + \frac{0.12}{1 - \left(\frac{0.494}{\lambda}\right)^2} - \frac{0.543}{\lambda} \right) \quad (4)$$

and $g = 0.8 - 0.004\lambda + 6.4 \cdot 10^{-6}\lambda^2 - 5 \cdot 10^{-9}\lambda^3 + 14 \cdot 10^{-13}\lambda^4$), ($\lambda$ in μm) which was obtained previously for a similar smooth film [35].

The obtained dependencies in Supplementary Figure 6 show a good correspondence with the experimental Fig.3 in the main text.

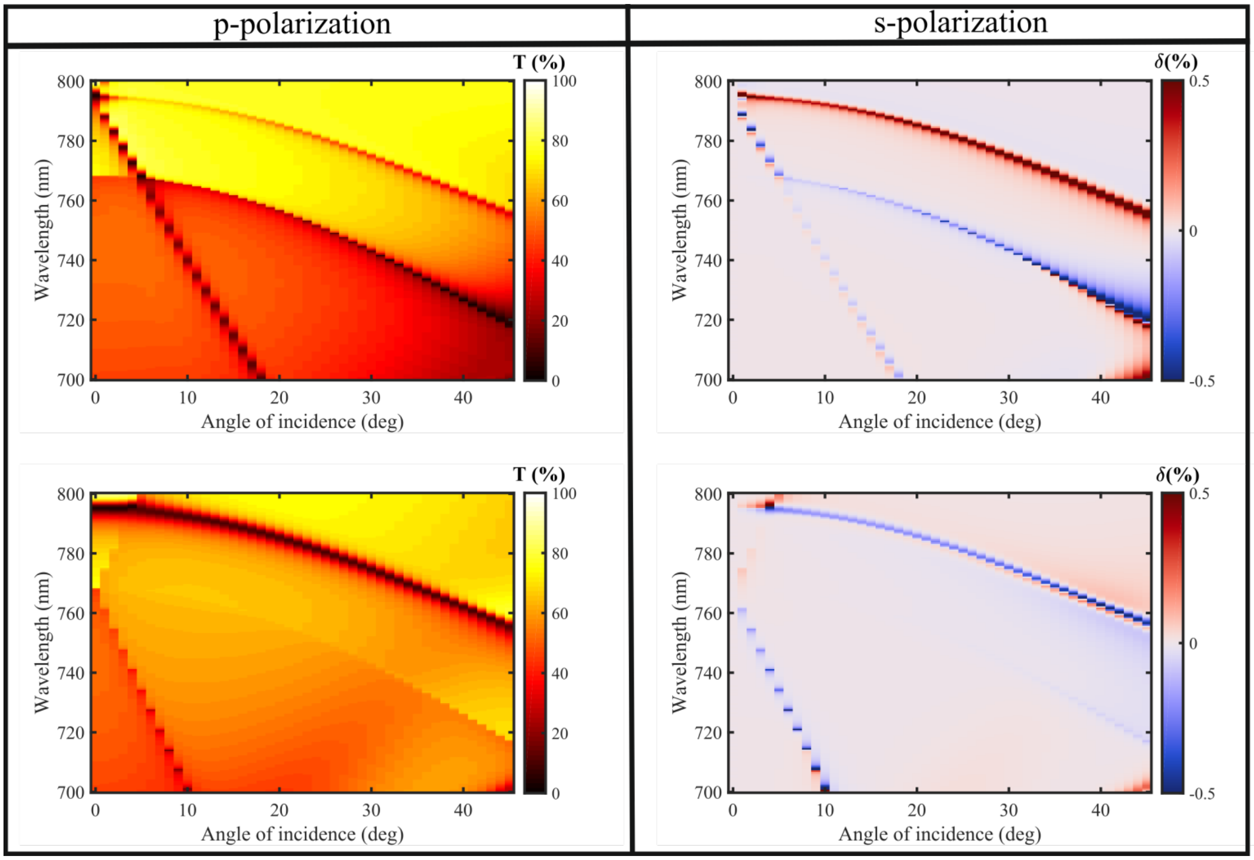

Supplementary Figure 6. Transmittance and magneto-optical intensity modulation spectra (numerical simulations).

[S1] M. G. Moharam, E. B. Grann, D. A. Pommet, T. K. Gaylord, J. Opt. Soc. Am. A, 12, 1068 (1995);
[S2] L. Li, J. Opt. A-Pure Appl. Op. 5, 345 (2003).
